# Female medical and nursing students’ knowledge, attitudes, and skills regarding breast self-examination in Oman: a comparison between pre- and post-training

**DOI:** 10.3352/jeehp.2020.17.37

**Published:** 2020-12-01

**Authors:** Rajani Ranganath, John Muthusami, Miriam Simon, Tatiyana Mandal, Meena Anand Kukkamulla

**Affiliations:** 1 Department of Pathology, College of Medicine & Health Sciences, National University of Science & Technology, Sohar, Oman; 2 Department of Surgery, College of Medicine & Health Sciences, National University of Science & Technology, Sohar, Oman; 3 Department of Psychology and Behavioral Sciences, College of Medicine & Health Sciences, National University, of Science & Technology, Sohar, Oman; 4 Department of Pharmacology, Melaka Manipal Medical College, Manipal, India; 5 Department of Periodontology, Melaka Manipal Medical College, Melaka, Malaysia; Hallym University, Korea

**Keywords:** Breast self-examination, Early detection of cancer, Medical students, Oman

## Abstract

**Purpose:**

Breast cancer is one of the most common cancers in women worldwide. Educational and awareness programs impact early practices of breast self-examination, resulting in the early detection of cancer and thereby decreasing mortality. The study aimed to assess the levels of knowledge and awareness of breast cancer and breast self-examination among medical and nursing students in Oman and to compare their knowledge, attitudes, and skills after a training program.

**Methods:**

This quasi-experimental study was carried out for female 90 medical and 80 nursing students in Oman in November 2019. A pre-test questionnaire was given before the training program and a post-test questionnaire was administered after the training program. Students’ knowledge, attitude, and skills regarding breast cancer and breast self-examination were compared. Scores for skills of practicing breast self-examination were compared between lecture and activity group and lecture-only group.

**Results:**

Pre-test and post-test data were collected from 170 female students. Significant improvements were observed in the post-test scores for students’ knowledge, attitude, and skills after the intervention (P<0.001). The mean scores for skills of practicing breast self-examination after the lecture and the activity were higher than those obtained after the lecture only (P=0.014 for medical students and P=0.016 for nursing students).

**Conclusion:**

An educational training program on breast cancer and breast self-examination with an emphasis on skills can motivate participants to perform breast self-examination regularly, and may therefore help students to train other women to perform breast self-examination for the early detection of breast cancer.

## Introduction

### Background/rationale

Breast cancer is one of the major cancers leading to morbidity and mortality among women in the Sultanate of Oman. According to the latest statistics released by the Ministry of Health, extending through 2015, it accounts for 26.9% of all cancer cases among women in the country [1]. One out of 5 Omani women is diagnosed with breast cancer in her lifetime and the overall standardized incidence rate is 15.6 cases per 100,000 [2]. The frequency of cancers among Omani women increased steadily from 64 in 2003 to 212 per 100,000 in 2015. Breast self-examination is a relatively simple, low-cost method of early detection that can be performed more frequently than mammography or clinical breast examinations. Monthly breast self-examination has been reported to be effective in detecting the early symptoms of breast cancer, and early detection in turn greatly reduces mortality from breast cancer. The implementation of breast cancer screening programs is strongly determined by national policies, healthcare organizations, available resources, and other factors. With the collaboration of various professionals in the health care sector and with the help of their expertise, it will be easier to achieve this common goal.

### Objectives

This study aimed to assess the level of knowledge and awareness of breast cancer and breast self-examination among medical and nursing students in Oman and to compare their knowledge, attitudes, and skills before and after a planned teaching program. Furthermore, it compared the effectiveness of the 2 educational intervention methods that were used (lecture versus active learning).

## Methods

### Ethics statement

Approval was obtained from the Research and Ethics Committee to conduct the study (CMHS/REC/038/19/C). Respondents participated in the survey and expressed their consent by completing an informed consent form.

### Study design

It is a quasi-experimental study carried out for female students (90 medical and 80 nursing students).

### Participants

Ninety female medical students (premedical and preclinical) and 80 female nursing students in their 1st year, 2nd year, and 3rd year participated in the program. Medical students in a clinical year, BSc nursing year 4 students, and students who did not provide consent to participate in the study were excluded. Medical students were from the College of Medicine and Health Sciences, National University of Science and Technology and nursing students were from Sohar Nursing Institute and North Batinah Nursing Institute in Oman.

### Study size

For the difference of means in a paired sample, a prior sample size was computed as 54 using G*Power ver. 3.1.9.4 (Heinrich-Heine-Universität Düsseldorf, Düsseldorf, Germany; http://www.gpower.hhu.de/), with the following assumptions: tails=2, effect size (d_z_)=0.5, α error probability=0.05, and power (1-β error probability)=0.95. Thus, the sample sizes of 80 and 90 in each group were sufficient.

### Educational intervention

The study was conducted in November 2019. The breast cancer awareness workshop was conducted for 2 days (Supplement 1). On each day of the program; there was a common lecture session, which was followed by a pre-test. Then the students were divided equally into 2 groups. Group 1 received a post-test immediately after the lecture session. The active learning session was done after the post-test, before the students were dismissed. Thus, the students were not deprived of the active learning. Group 2 had an active learning session (video demonstration of breast self-examination, hands-on activity using a breast model for examining the breast, and clay activity to determine different tumor sizes) after the lecture session. This was followed by a post-test. For the pre- and post-test, self-administered questionnaires were given. The breast model used in the program was developed by the principal investigator and was approved by subject experts.

### Measurement tool

To develop the questionnaire, an extensive review of the literature was done and items were developed in reference to already validated items in published articles written by Ahmed et al. [3] and Madhukumar et al. [4]. The tools of both articles were used under the Creative Commons license without the permission of the authors. A questionnaire containing 24 items was developed that addressed different variables on knowledge of breast cancer and knowledge of, attitude towards, and skills of breast self-examination (Supplement 2). The following 4 domains were included in the questionnaire: knowledge of breast cancer (7 items), knowledge of breast self-examination (5 items), attitudes towards breast self-examination (8 items), and skills of practicing breast self-examination (4 items). Correct answers for knowledge of breast cancer and breast self-examination were marked as 1 and incorrect answers were marked as 0, upon basis the total scores were calculated. For the attitude items, a 5-point Likert scale (strongly agree, agree, neutral, disagree, strongly disagree) was used. For positive-attitude items, scores of 5, 4, 3, 1, and 1 corresponded to “strongly agree,” “agree,” “neutral,” “disagree,” and “strongly disagree,” respectively. This scoring was reversed for negative-attitude items. The questionnaire also included a few subjective-type questions on information and practices of performing a breast self-examination.

### Validity and reliability test of the measurement tools

The content validity of the questionnaire was checked by 5 experts from the College of Medicine and Health Sciences, National University of Science and Technology, Oman. Each expert was given a copy of the scale and received an individual explanation of the purpose and objectives of the study. The experts were then asked to rate each item based on relevance, clarity, grammar/spelling, ambiguity, and structure of the sentences on a 5-point Likert scale. The item-level content validity index (I-CVI), scale’s content validity index (S-CVI), and modified kappa were analyzed for all the questions using Microsoft Excel (Microsoft Corp., Redmond, WA, USA). The items that had a CVI over 0.75 remained and those ranged from 0.70 and 0.79 were revised. The S-CVI calculated using universal agreement (UA) was calculated by adding all items with an I-CVI equal to 1 divided by the total number of items. The S-CVI/UA of 24 items ranged between ≥0.8 and ≥0.9, meaning that the items had excellent content validity. The modified kappa was calculated using the formula: kappa= (I-CVI-Pc)/ (1-Pc), where Pc = [N!/A!(N-A)!]*0.5^N^. In this formula Pc = the probability of chance agreement; N = number of experts; and A = number of experts that agree the item is relevant. Items with a kappa value ≥0.74 were retained and those with values ranging from 0.04 to 0.59 were revised. The teaching module was validated by a panel of experts. The reliability of Likert-type questions was tested and the Cronbach α was 0.765.

### Statistical methods

Descriptive data are presented as frequency and percentage, while continuous data are presented as mean and standard deviation. The pre- and post-scores for knowledge of breast cancer and knowledge, attitudes, and skills regarding breast self-examination were compared using the paired t-test. The post-test skills of practicing breast self-examination (after lecture only and after lecture and activity) were compared using the Student t-test. Data were analyzed using IBM SPSS ver. 22.0 (IBM Corp., Armonk, NY, USA).

## Results

### Participants

Eighty-six students were between 18 and 20 years of age, while 84 were above 20 years of age. Of the medical students, 21, 16, 21, and 32 were in years 2, 3, 4, and 5 of the curriculum, respectively. Of the nursing students, 29, 32, and 19 were in years 1, 2, and 3 of the BSc in nursing program, respectively (Table 1).

### Main results

The raw data of the students’ responses are available in Datasets 1 and 2. A statistically significant improvement was observed in the post-test scores for knowledge, attitudes, skills after the intervention (P<0.001). The mean pre- and post-test scores for the 4 domains (knowledge of breast cancer and knowledge, attitudes, and skills regarding breast self-examination) are presented in Tables 2 and 3. The mean scores for attitudes showed no significant difference between the pre-test and post-test among nursing students (P=0.320).

The mean scores for skills of practicing breast self-examination after the lecture and active learning were 5.69 and 5.45, respectively, for medical and nursing students, whereas the mean scores obtained after the lecture only was 5.33 and 4.95, respectively. These results were statistically significant (P=0.014 and P=0.016, respectively).

#### Knowledge of breast self-examinations

Seventy-two of the 90 medical students and 57 of the 80 nursing students had heard about breast self-examination before the training program. Most of the students received information through doctors (24 medical students), the internet (21 medical students), and family members (24 nursing students).

#### Skill of practicing breast self-examination

**Fig. 1.**
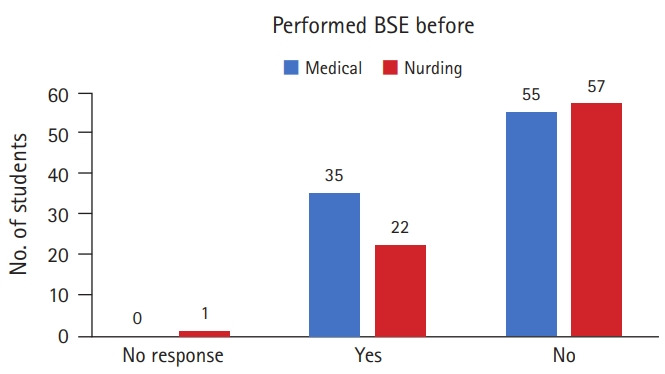
Number of students who performed BSE before the training program. BSE, breast self-examinations.

Only 35 medical students and 22 nursing students had performed breast self-examination before (Fig. 1). Some students had performed breast self-examination based on advice they had received from family members (17 medical students) and doctors (13 medical and 16 nursing students). Furthermore, 24 medical students and 36 nursing students did not know how to perform breast self-examination.

## Discussion

### Interpretation

The breast cancer awareness program was successful in fostering awareness of breast cancer and breast self-examination among medical and nursing students. The students’ prior knowledge of breast cancer and breast self-examination was assessed before the implementation of the training program. The pre-test findings demonstrated low levels of knowledge in all the areas tested. This gap in students’ knowledge among students is not enough. This result is in agreement with those reported by Kıssal et al. [5] in 2017. Significant improvements in the knowledge of breast cancer and self-examination were observed after the training program. These improvements can be attributed to the content of the program, which covered all the identified needs and knowledge gaps among the students. Likewise, an educational intervention on breast cancer screening practice uptake, knowledge, and health beliefs among Yemeni female school teachers in Klang Valley, Malaysia was proven to be effective in a randomized controlled trial [6].

The media play a significant role in creating awareness and can therefore be used as the main source of information about breast cancer and to promote breast self-examination [7]. In our study, the students come from educated family backgrounds; hence, their source of information were mainly through doctors (24 medical students), the internet (21 medical students), and family members (24 nursing students).

In the present study, attitudes towards breast cancer and breast self-examination appeared to be positive among the majority of the students after the intervention. This training program motivated the students, as shown by improvements in the mean scores in the post-test. However, the mean pre-test and post-test scores for attitudes among nursing students did not show a statistically significant difference. Videos on experiences shared by breast cancer survivors were used to motivate students regarding the importance of early detection of breast cancer. However, newer teaching methods can be experimented with to impart positive attitudes. Therefore, it is important to explore newer ideas to motivate students to practice breast self-examination regularly. The study conducted by Kıssal and Kartal [8] in 2019 showed the effectiveness of an educational intervention in improving attitudes among nursing students towards breast self-examination.

The findings of the current study revealed that the students did not have sufficient knowledge and skills to correctly perform the steps of breast self-examination before the intervention. The reasons for this could be a lack of awareness, training, and motivation. A similarly low prevalence of practicing breast self-examination was reported in a study from Ajman in the United Arab Emirates [9]. However, the post-test of the present study demonstrated significant improvements in students’ performance of breast self-examination, which can be clearly attributed to the skills activity, which involved a demonstration of all the steps of a breast self-examination using breast model, a video demonstration of breast self-examination, and a clay activity to determine and recognize different tumor sizes. Each student was given an opportunity to demonstrate the breast model and clay activity, which facilitated a positive learning environment and helped them develop confidence of in performing breast self-examination. The effectiveness of the program in improving the practice of breast self-examination aligns with a number of studies [10, 11]. Creating an exciting learning setting and using a breast model to teach is more impactful than just giving didactic lectures, as demonstrated in our study. The mean scores for skills of practicing breast self-examination after the lecture and the activity were higher than those obtained after the lecture only (P=0.014 and P=0.016 for medical and nursing students respectively). Hence the present intervention, which taught skills and knowledge, was helpful for better understanding and retaining the content of the study.

### Limitations

The study was conducted only for premedical and preclinical students and BSc nursing students in years 1, 2, and 3, who had no clinical exposure.

### Generalizability

Breast cancer is a global concern; each and every country has breast cancer awareness programs. Implementation of effective methods of teaching breast self-examination would motivate women around the world to practice breast self-examination regularly.

### Conclusion

An important dimension in breast cancer awareness is to inculcate innovative techniques to create interest and motivate a positive attitude for performing a breast self-examination. All universities with degree programs in the medical or other allied health professions can initiate such programs for their students. Promoting this kind of educational intervention will have a positive impact in spreading awareness on breast cancer for early detection for better survival and health outcomes.

## Figures and Tables

**Table 1.** Demographic information of the surveyed students

Characteristic	Category	No. (%)
Age (n=170)	18–20 yr	86 (51)
	>20 yr	84 (49)
Marital status (n=170)	Married	3 (2)
	Unmarried	167 (98)
Academic year	Medical (n=90)	
	Year 2	21 (23)
	Year 3	16 (18)
	Year 4	21 (23)
	Year 5	32 (36)
	Nursing (n=80)	
	Year 1	29 (36)
	Year 2	32 (40)
	Year 3	19 (24)
Family history of breast cancer	Yes	14 (8)
	No	156 (92)
If yes, the relationship (n=14)	Aunt	9 (64)
	Sister	4 (29)
	Grandmother	1 (7)

**Table 2.** Comparison of pre- and post-test scores after the program on breast self-examination among medical and nursing students in Oman

Variable	Medical			Nursing		
	Pre-test	Post-test	P-value	Pre-test	Post-test	P-value
Knowledge of breast cancer	11.6±2.6	16.8±2.2	<0.001	8.2±3.0	14.2±2.4	<0.001
Knowledge of breast self-examination	1.9±1.2	2.8±0.6	<0.001	1.6±1.3	2.6±0.6	<0.001
Attitudes towards breast-self-examination	29.2±4.2	31.1±3.8	0.002	27.4±4.3	28.1±4.3	0.32

Values are presented as mean±standard deviation. Maximum scores: knowledge of breast cancer, 22; knowledge of breast self-examination, 3; attitudes, 40.

**Table 3.** Skills of practicing breast self-examination (pre-test and post-test) among medical and nursing students

Skills	Group 1^a)^			Group 2^b)^		
	Pre-test	Post-test	P-value	Pre-test	Post-test	P-value
Medical	3.42±0.988	5.33±0.769	<0.001	3.76± 1.368	5.69±0.557	<0.001
Nursing	2.65±1.424	4.95±1.085	<0.001	3.00±1.633	5.45±0.677	<0.001

Values are presented as mean±standard deviation. Maximum score for skills: 6.

a) Aafter lecture only.

b) After lecture and activity.

## Footnotes

**Data availability** Data files are available from Harvard Dataverse: https://doi.org/10.7910/DVN/UAOMR7 Dataset 1. Raw data of the students’ responses on the pre-test before the educational intervention. Dataset 2. Raw data of the students’ responses on the post-test after the educational intervention.

## Supplementary materials

Data files are available from Harvard Dataverse: https://doi.org/10.7910/DVN/UAOMR7

Supplement 1. Schedule of the breast cancer awareness program done November 11 and 12, 2019 at the National University of Science & Technology, Sohar, Oman.

Supplement 2. The 24-item questionnaire used in this study.

Supplement 3. Audio recording of the abstract.
