# Supplementary material for: Female medical and nursing students’ knowledge, attitudes, and skills regarding breast self-examination in Oman: a comparison between pre- and post-training
Source: J Educ Eval Health Prof. 2020 Dec 1;17:37. doi: 10.3352/jeehp.2020.17.37 (PMC7803588; doi:10.3352/jeehp.2020.17.37)
Supplement: Supplementary file 3 — Supplement 1. Schedule of the breast cancer awareness program done November 11 and 12, 2019 at the National University of Science & Technology, Sohar, Oman. [file jeehp-17-37-suppl1.pdf]

College of Medicine & Health Sciences
Tel +968 22 31 4700, P.O. Box 620, PC 130
Sohar Campus, Sultanate of Oman
Email: info@nu.edu.om | Website: www.nu.edu.om

الجامعة الوطنية
National University
Science & Technology للعلوم والتكنولوجيا

Breast Cancer Awareness Workshop
An Interprofessional Education Initiative

Date: 12th November 2019
Time: 3:00 pm – 5:00pm
Venue: College of Medicine and Health Sciences,

Program

Time	Topics	Speaker
**Lecture Sessions**		
3.00- 3.05pm	Welcome by Dean	Prof. Mohammed Al Shafaee- Dean
3:05-3:15 pm	Introduction	Dr Rajani Ranganath
3.15 – 3.20 pm	My story	Video
3.20- 3.35pm	Risk factors & benign lumps	Dr Rajani Ranganath
3.35- 3.50pm	Myths and Facts about breast cancer	Dr Miriam Simon
3.35- 3.50pm	Breast cancer Clinical presentation and self- examination	Dr John Muthusami
3.50-4.05pm	Role of the nurse in breast care practice and breast cancer awareness	Nursing Faculty
**Active Learning Sessions – Audio Visual & Hands on**		
**Students are divided in to small groups for activity**		
4.05-4.30 pm	• Breast self-examination video<br>• Hands on activity of breast self-examination using breast model<br>• Clay Activity<br><br>Students Groups are formed (5 students = 1group)	All faculty and staff
4.30 -4.45pm	Closing Remarks	

الـجامـعـة الوطـنيـة
National University
Science & Technology للعلـوم والتكنــولوجيا

BREAST CANCER AWARENESS WORKSHOP

AN INTERPROFESSIONAL EDUCATION INITIATIVE

Touch to feel the difference

Outubro ROSA

College of Medicine & Health Sciences

Oman College of Health Sciences

Date: 11-12/ November 2019

Venue: College of Medicine – Amphitheater B (Big Lecture Hall)
