# Supplementary material for: Female medical and nursing students’ knowledge, attitudes, and skills regarding breast self-examination in Oman: a comparison between pre- and post-training
Source: J Educ Eval Health Prof. 2020 Dec 1;17:37. doi: 10.3352/jeehp.2020.17.37 (PMC7803588; doi:10.3352/jeehp.2020.17.37)
Supplement: Supplementary file 4 — Supplement 2. The 24-item questionnaire used in this study. [file jeehp-17-37-suppl2.docx]

**Informed consent for participation in the study;**

M /N - _______

This questionnaire has been designed as a part of research study looking at different teaching and learning methods. Your participation in this research study is voluntary. Questionnaire filling up will take approximately 5 minutes. Response/s received by the participants will be kept confidential .You are required to complete all fields of the questionnaire, the data of which will be under protection of principal investigator. The results of this study will be used only for research purpose.

**Signature: _____________**

**Questionnaire on awareness of breast cancer and Breast self-examination**

**Demographic information:**

Age (Yrs.):

Married/Unmarried:

Education status: Medical – MD 1, MD 2

Medical – MD 3, MD 4

Nursing - Year of course ________

Family history of breast cancer a) yes b) no

If **yes** mention the relationship: ________________________

**Knowledge on breast cancer**

1. The chances of getting breast cancer is less in which of the following situation:
2. Single relative diagnosed with both breast and ovarian cancer
3. Family members who develop cancer in both breasts
4. Male relative diagnosed with breast cancer
5. Breast cancer that developed after age of 50 years in a family member
6. The lowest age that breast cancer can occur is as early as in the :-
7. 15 -19yrs b) 20 -25 years c)26 -30 4)30 -35 years
8. Tick risk factors that can cause breast cancer from the list given below :( Can give multiple answers)

| **Risk Factor** | **Tick (🗹 )** |
| --- | --- |
| Increasing age |  |
| Early menarche and late menopause |  |
| Women with dense breasts |  |
| Null parity (women who has never become pregnant ) |  |
| First pregnancy before 30yrs of age |  |
| Inherited mutation of BRCA 1&2 gene |  |
| Breast trauma |  |
| Exclusive breast feeding |  |
| Alcohol |  |

1. Tick the warning signs of breast cancer from the table given below: ( Can give multiple answers)

| **Signs of breast cancer** | **Tick (🗹 )** |
| --- | --- |
| Hard lump in the breast or armpit |  |
| Swelling and redness of the breast |  |
| Size of both breast are uneven |  |
| Bloody discharge from nipple |  |
| Pus discharge from the nipple |  |
| Nipples are everted |  |
| Nipples are retracted |  |
| Dimpling of the skin |  |

1. Which of the following is screening methods is suitable for pregnant women to detect breast cancer?
2. Mammogram
3. Ultrasound
4. MRI
5. Chest X-ray
6. At what age should mammogram screening be started for the general population?
7. 40years b) 50 years c) 45 years d) 35 years
8. Annual mammogram screening in the general population is done after _______ years.(Fill in the blank)

**Knowledge of Breast self-examination (BSE)**

1. Have you heard of BSE before a) yes b) no
2. If yes, from where?
3. Doctor b) Books c) Internet d) Family members /friends e) others specify __________
4. At what age should BSE be started?
5. From 20 years (b) From 30 years (c) from 40 years d) after menopause

1. How often should BSE be done?

1. Weekly (b) Monthly c) Yearly d) Daily
2. What is the appropriate time to perform BSE?

a) A week before menstruation

b) A week after menstruation

c) During ovulation

d) Anytime

**Attitude towards Breast self-examination (BSE)**

1. What I feel about BSE; Tick the appropriate option

| 1.I feel it is important to learn BSE | Strongly  Agree | Agree | Neutral | Disagree | Strongly  Disagree |
| --- | --- | --- | --- | --- | --- |
| 2.I fear/ feel uncomfortable going to a physician for a checkup if I find a lump | Strongly  Agree | Agree | Neutral | Disagree | Strongly  Disagree |
| 3.I avoid BSE for fear of consequences of breast cancer | Strongly  Agree | Agree | Neutral | Disagree | Strongly  Disagree |
| 4.I feel uncomfortable to examine my breasts | Strongly  Agree | Agree | Neutral | Disagree | Strongly  Disagree |
| 5.There are not enough awareness programs in our community to teach BSE | Strongly  Agree | Agree | Neutral | Disagree | Strongly  Disagree |
| 6.If you do a routine mammography, you do not need to perform BSE | Strongly  Agree | Agree | Neutral | Disagree | Strongly  Disagree |
| 7.If you have a breast examination performed by a healthcare worker, you do not need to perform BSE | Strongly  Agree | Agree | Neutral | Disagree | Strongly  Disagree |
| 8.I encourage BSE for my family and community members | Strongly  Agree | Agree | Neutral | Disagree | Strongly  Disagree |

**Skills of practicing** **Breast self-examination (BSE)**

1. Have you ever performed BSE before?

Yes

No

1. If yes, what was the purpose for performing it?

Advice from friends and family

Advice from doctor

You noticed a breast lump

Family history of breast cancer

1. If no, why haven’t you performed it?

Fear of detecting a lump

Not necessary as I am under 20 years

Too busy

Don’t know how to do self‑examination

1. How do you perform BSE- Tick the correct option

| 1. What is the ideal way to inspect the breast? | Standing in-front of the mirror | While bathing | While sleeping |
| --- | --- | --- | --- |
| 1. How do you palpate the breast? | Using thumb and index finger | Using middle three finger | Whole palm |
| 1. Which hand do you use for BSE? | hand of same side of the breast | Hand of the opposite side of breast | Use single hand for both the breast |
| 1. Which is **NOT** the correct method to evaluate nipple discharge? | Pressing it with the thumb and index finger | By observing it. | By blotting with the paper |
| 1. Where else do you look for a lump other than the examining breast? | Armpit | Neck | Back |
| 1. Which of the following is **NOT** the technique for performing BSE? | Circular | Vertical | Triangular |
